# New tardigrade records for the Baltic states with a description of *Minibiotus formosus* sp. n. (Eutardigrada, Macrobiotidae)

**DOI:** 10.3897/zookeys.408.6612

**Published:** 2014-05-13

**Authors:** Krzysztof Zawierucha, Jakub Dziamięcki, Natalia Jakubowska, Łukasz Michalczyk, Łukasz Kaczmarek

**Affiliations:** 1 Department of Animal Taxonomy and Ecology, Faculty of Biology, A. Mickiewicz University, Umultowska 89, 61-614 Poznań, Poland; 2 Department of Water Protection, Faculty of Biology, A. Mickiewicz University, Umultowska 89, 61-614 Poznań, Poland; 3 Department of Entomology, Institute of Zoology, Jagiellonian University, Gronostajowa 9, 30-387 Kraków, Poland

**Keywords:** Estonia, Europe, *Hypsibius* cf. *scabropygus*, Latvia, Lithuania, new species, Tardigrada

## Abstract

In sixteen moss, lichen and mixed (moss/lichen) samples, collected from Estonia, Latvia and Lithuania, 291 specimens, 48 simplexes, including one exuvium with 6 eggs, and 8 free-laid eggs of eutardigrades were found. In total, 17 species, together with one new to science, were identified (all are new records for the Baltic states): *Astatumen bartosi*, *Diphascon (Adropion) prorsirostre*, *D. (Diphascon) bullatum*, *D. (D.) pingue pingue*, *D. (D.) recamieri*, *D. (D.) rugosum*, *Hypsibius convergens*, *H. dujardini*, *H.* cf. *scabropygus*, *Isohypsibius ronsisvallei*, *I. sattleri*, *Macrobiotus harmsworthi harmsworthi*, *M. hufelandi hufelandi*, *Milnesium asiaticum*, *Milnesium tardigradum tardigradum*, *Minibiotus formosus* **sp. n.** and *Paramacrobiotus richtersi*. The new species is most similar to *Minibiotus gumersindoi*, but differs from it mainly by the presence of two types of cuticular pores, the absence of a triangular or pentagonal arrangement of pores above a single large pore on legs, the presence of granulation on all legs and a different macroplacoid length sequence. In this paper we also provide photographs and morphometrics of *H.* cf. *scabropygus*.

## Introduction

The Baltic States, i.e. Estonia, Latvia and Lithuania, are located on the eastern coast of the Baltic Sea, and fall within the Palearctic ecozone (Holt et al. 2012). The topography of the three countries is dominated by lowlands with the highest peaks at *ca.* 300 m asl. The temperate climate is intermediate between maritime and continental. Even though the phylum Tardigrada is cosmopolitan and currently comprises *ca.* 1,200 species (Degma et al. 2013), so far only six tardigrade taxa have been reported from the Baltic States. Specifically, two from Estonia: *Eremobiotus alicatai* (Binda, 1969) and *Isohypsibius* cf. *marcellinoi* (Binda & Pilato, 1971), two from Latvia: *Paramacrobiotus richtersi* group and *Macrobiotus hufelandi* group and two from Lithuania: *Macrobiotus* sp. and *Ramazzottius* sp. (Šatkauskienė and Vosyliūtė 2010, Zawierucha and Kaźmierski 2012, Ziemelis et al. 2012).

In this study we report seventeen tardigrade species, which are all new records for the Baltic States. Moreover, one of these species is also new to science. The new species belongs to the genus *Minibiotus* R.O. Schuster, 1980, that until 1988 contained only a single species, *Minibiotus intermedius* (Plate, 1888). In 1988 Pilato and Claxton (1988) described *Minibiotus maculartus*, and within the last decade fourteen new *Minibiotus* species have been described. Several species have also been transferred to *Minibiotus* from the genus *Macrobiotus* based on characters defined by Claxton (1998) and later supplemented by Guidetti et al. (2007) (Michalczyk and Kaczmarek 2003a, Pilato et al. 2003, Michalczyk and Kaczmarek 2004, Guil and Guidetti 2005, Michalczyk et al. 2005, Pilato and Lisi 2006b, Li et al. 2008, Fontoura et al. 2009a, b, Rossi et al. 2009, Meyer and Hinton 2009, Meyer and Domingue 2011, Meyer et al. 2011 Meyer 2012). Currently, the total number of *Minibiotus* species amounts to as many as forty seven.

In addition to the description of the new species, we also provide morphometric data and photographs of *Hypsibius* cf. *scabropygus*, a rare species that belongs to a large group of hypsibiids with at least partially sculptured dorsal cuticle and pharynx with two macroplacoids and without the microplacoid.

## Material and methods

Sixteen moss, lichen and mixed (moss/lichen) samples from trees, soil and stones were collected from 15 localities in Estonia, Latvia and Lithuania between the 29 April and the 5 May 2012 by the third author (more details below). Samples were collected and examined for tardigrades using standard methods (see Dastych 1980). After extraction, animals were mounted on microscope slides in Hoyer’s medium. All specimens were examined measured and photographed using Phase Contrast Microscopy (PCM) or Scanning Electron Microscopy (SEM). In total 358 specimens (including 47 simplexes), one exuvium with 6 eggs, and 8 free-laid eggs were examined.

All measurements are given in micrometers [μm]. Structures were measured only if their orientation was appropriate. Body length was measured from the anterior extremity to the end of the body, excluding the hind legs. Buccal tube length and the level of the stylet support insertion point were measured according to Pilato (1981). Buccal tube width was measured as the external diameter at the level of the stylet support insertion point. Lengths of the claw branches were measured from the base of the claw to the top of the branch including accessory points for *Minibiotus* and according to Beasley et al. (2008) for *Hypsibius*. The *pt* ratio is the ratio of the length of a given structure to the length of the buccal tube expressed as a percentage (Pilato 1981). Macroplacoid length sequence is given according to Kaczmarek et al. (2014b), i.e. macroplacoids are listed from the shortest to the longest and their relative sizes are denoted with appropriate inequality, approximation and/or equality signs (<, ≤, ≈, =). Morphometric data were handled using the ‘Macrobiotoidea’ ver. 1.1 template available from the Tardigrada Register (www.tardigrada.net/register, Michalczyk and Kaczmarek 2013).

For species identification and differentiation, keys in Claxton (1998), Fontoura and Pilato (2007), Kaczmarek et al. (2011), Michalczyk et al. 2012a, b and Ramazzotti and Maucci (1983), and original descriptions and redescriptions (Ehrenberg 1859, Ramazzotti 1959, 1962, Horning et al. 1978, Bertolani and Rebecchi 1993, Dastych 1988, 1990, Binda and Pilato 1992, Michalczyk and Kaczmarek 2004, Michalczyk et al. 2005, Miller et al. 2005, Fontoura et al. 2009a, b, Meyer and Hinton 2009, Meyer et al. 2011) as well as for insertion of the stylet muscles Pilato (2013) were used. Tardigrade taxonomy is presented according to Marley et al. (2011). Only specimens determined to species level are provided in the list of species (we omitted all specimens determined only to the species group level, e.g. the *hufelandi* group or the *oberhaeuseri* group). In the species list Roman numbers indicate sample codes (see sampling localities) and Arabic numbers indicate the number of specimens, exuvia/simplexes and eggs.

Raw data underlying the description of *Minibiotus formosus* sp. n. are deposited in the Tardigrada Register (Michalczyk and Kaczmarek 2013) under www.tardigrada.net/register/0012.htm.

### Sampling localities

- 56°03'08"N, 24°24'10"E, *ca.* 33 m asl: Lithuania, Panevėžys county, Pasvalys district municipality, along the road E67, 0.5 km before the turning to Pasvalys, moss from tree and soil (slide code: LT 2422), date: 29.04.2012.
- 55°25'59"N, 24°13'32"E, *ca.* 59 m asl: Lithuania, Kaunas county, Kėdainiai district municipality, Truskava city, near a church, lichens from tree and moss from soil (slide code: LT 2423), date: 29.04.2012.
- 55°17'12"N, 23°58'57"E, *ca.* 30 m asl: Lithuania, Kaunas county, Kėdainiai district municipality, Kėdainiai city, Kranto II street; moss from wall (slide code: LT 2424), date: 29.04.2012.
- 55°17'13"N, 23°58'56"E, *ca.* 30 m asl: Lithuania, Kaunas county, Kėdainiai district municipality, Kėdainiai city, Paeismilgio street; moss from stone (slide code: LT 2425), date: 29.04.2012.
- 55°43'35"N, 24°21'30"E, *ca.* 62 m asl: Lithuania, Panevėžys county, Panevėžys district municipality, Panevėžys city, Garden Street near Holy Trinity Rector; moss from tree (slide code: LT 2440), date: 05.05.2012.
- 56°38'53"N, 23°43'18"E, *ca.* 7 m asl: Latvia, Zemgale region, Jelgava municipality, Jelgava city, City Park; moss from soil (slide code: ŁO 2426), date: 29.04.2012.
- 57°10'33"N, 24°50'32"E, *ca.* 45 m asl: Latvia, Vidzeme region, Sigulda municipality, Gutmana Cave in the Gauja National Park; moss from rocks (slide code: ŁO 2427), date: 30.04.2012.
- 56°23'55"N, 24°07'33"E, *ca.* 25 m asl: Latvia, Zemgale region, Bauska municipality, along Road No P103, 0.5 km from Saulaine; lichens from tree (slide code: ŁO 2428) date: 29.04.2012.
- 57°09'55"N, 24°51'03"E, *ca.* 73 m asl: Latvia, Vidzeme region, Sigulda municipality, Turaida city, Turaida Castle; moss from stone (slide code: ŁO 2430), date: 30.04.2012.
- 56°54'32"N, 24°08'45"E, *ca.* 10 m asl: Latvia, Riga Region, boundary of Ķekava municipality, along road no A2; moss from tree (slide code: ŁO 2431), date: 30.04.2012.
- 57°09'59"N, 24°50'59"E, *ca.* 91 m asl: Latvia, Vidzeme region, Sigulda municipality, Sigulda city, Sigulda Castle; moss from stone (slide code: ŁO 2432), date: 30.04.2012.
- 56°41'22"N, 23°47'43"E, *ca.* 4 m asl: Latvia, Zemgale region, Ozolnieki municipality, Ozolnieki city, about 100 m from the Ozolnieki Lake; moss from soil (slide code: ŁO 2433), date: 29.04.2012.
- 57°45'43"N, 24°20'59"E, *ca.* 3 m asl: Latvia, Vidzeme region, Salacgriva municipality, Salacgriva city; moss from soil, near the beach (slide code: ŁO 2434), date: 01.05.2012.
- 59°10'44"N, 24°30'06"E, *ca.* 59 m asl: Republic of Estonia, Harju county, Kernu Parish municipality, Road No 4, moss from tree (slide code: ES 2420), date: 04.05.2012.
- 59°10'44"N, 24°30'06"E, *ca.* 59 m asl: Republic of Estonia, Harju county, Kernu Parish municipality, Road No 4, moss from tree (slide code: ES 2421), date: 04.05.2012.
- 58°48'47"N, 24°24'46"E, *ca.* 32 m asl: Republic of Estonia, Rapla County, Märjamaa municipality, forest near Konuvere village, moss from tree (slide codes: ES 2487), date: 29.04.2012.

## Results

### Taxonomic accounts of species found in the study

#### Phylum: Tardigrada (Spallanzani, 1777)

##### Class: Eutardigrada Richters, 1926

###### Order: Apochela Schuster, Nelson, Grigarick and Christenberry, 1980

####### Family: Milnesiidae Ramazzotti, 1962

######## Genus: *Milnesium* Doyère, 1840

######### Milnesium asiaticum

Tumanov, 2006

########## Localities and specimen numbers.

XV: 1 specimen.

########## Remarks.

Our specimen corresponds perfectly to the original description. *Milnesium asiaticum* was originally described from Kirghizstan and subsequently found in the Svalbard archipelago (Tumanov 2006, Kaczmarek et al. 2012).

######### Milnesium tardigradum tardigradum

Doyère, 1840

########## Localities and specimen numbers.

VIII: 31 specimens (including 6 simplexes) + 1 exuvium with 6 eggs.

########## Remarks.

Specimens correspond perfectly with the redescription by Michalczyk et al. (2012a, b). This species was reported from many localities throughout the World, however records prior to Michalczyk et al. (2012a, b) need to be verified. So far, all confirmed localities are exclusively European (Michalczyk et al. 2012a, b).

###### Order: Parachela Schuster, Nelson, Grigarick & Christensen, 1980

####### Superfamily: Hypsibioidea Pilato, 1969 (in Marley et al. 2011)

######## Family: Hypsibiidae Pilato, 1969

######### Subfamily: Diphasconinae Dastych, 1992

########## Genus: *Diphascon* Plate, 1888

########### Subgenus: *Diphascon (Diphascon)* Pilato, 1987

############ Diphascon (Diphascon) bullatum

Murray, 1905

############# Localities and specimen numbers.

XIII: 1 specimen.

############# Remarks.

Specimens correspond well with the limited original description (Murray 1905) and also with later descriptions (Argue 1974 and Dastych 1980, 1988). This species is very similar to *Diphascon (Diphascon) patanei* (Binda & Pilato, 1971) and, as suggested by Dastych (1988), these two species could be synonymous. Thus, to clarify the taxonomic status of *Diphascon (Diphascon) patanei*, a re-description of *Diphascon (Diphascon) bullatum* is necessary based on material from *locus typicus* in Scotland (the type material probably does not exist) (Dastych 1988).

############ Diphascon (Diphascon) pingue pingue

(Marcus, 1936)

############# Localities and specimen numbers.

XV: 11 specimens.

############# Remarks.

Although we have found only 11 specimens, we were confident in identifying them to *Diphascon (Diphascon) pingue* because they corresponded perfectly to the partial redescriptions by Pilato and Binda (1997/1998, 1999) and we also identified them with the key by Fontoura and Pilato (2007). The species belongs to the *pingue* group and has been previously recorded from numerous localities throughout the World, however the majority of records should be verified based on the modern taxonomy (Pilato and Binda 1997/1998, 1999). Currently, exclusively verified localities of this species are only from Europe and North America (Pilato and Binda 1997/1998).

############ Diphascon (Diphascon) recamieri

Richters, 1911

############# Localities and specimen numbers.

XV: 1 specimen.

############# Remarks.

The species has previously been found in many localities, mostly in the Holarctic (McInnes 1994).

############ Diphascon (Diphascon) rugosum

(Bartoš, 1935)

############# Localities and specimen numbers.

II: 3 specimens.

############# Remarks.

The species has previously been found in many localities in the Holarctic (McInnes 1994).

########## Genus: *Diphascon* Pilato, 1987

########### Subgenus Diphascon (Adropion) prorsirostre

Thulin, 1928

############ Localities and specimen numbers.

XIV: 2 specimens, XV: 2 specimens.

############ Remarks.

The species has been previously found in many localities, mostly in the Holarctic (McInnes 1994).

######### Subfamily: Hypsibiinae Pilato, 1969

########## Genus: *Hypsibius* Ehrenberg, 1848

########### Hypsibius convergens

(Urbanowicz, 1925)

############ Localities and specimen numbers.

IX: 6 specimens (including 2 simplexes).

############ Remarks.

Belonging to the cosmopolitan *convergens*-*dujardini* complex of species (McInnes 1994, Miller et al. 2005, Kaczmarek and Michalczyk 2009a, Kaczmarek et al. 2014a), *Hypsibius convergens* used to be considered cosmopolitan, but it is most likely a complex of very similar (possibly also cryptic) species found throughout the world. The original *Hypsibius convergens* description no longer conforms to modern standards and therefore a redescription is required. Nevertheless, the examined specimens correspond perfectly with the original description and the *Hypsibius convergens* characteristics reviewed by Miller et al. (2005).

########### Hypsibius dujardini

(Doyère, 1840)

############ Localities and specimen numbers.

XIV: 2 specimens (including 1 simplex), XV: 5 specimens.

############ Remarks.

*Hypsibius dujardini* belongs to the cosmopolitan *convergens*-*dujardini* complex of species (McInnes 1994, Miller et al. 2005, Kaczmarek and Michalczyk 2009a, Kaczmarek et al. 2014a), and used to be considered cosmopolitan, but it is most likely a complex of similar (possibly cryptic) species found throughout the world. Due to the limited original description, *Hypsibius dujardini* needs a modern redescription. Nevertheless, the examined specimens correspond perfectly with the original description and the *Hypsibius dujardini* characteristics reviewed by Miller et al. (2005).

########### Hypsibius cf. scabropygus

Cuénot, 1929

Table 1 , Figs 1 –7

############ Localities and specimen numbers.

XI: 1 simplex, XII: 1 specimen, XIV: 34 specimens (including 4 simplexes), XV: 24 specimens (including 6 simplexes).

############ Description

**Figures 1–5.**
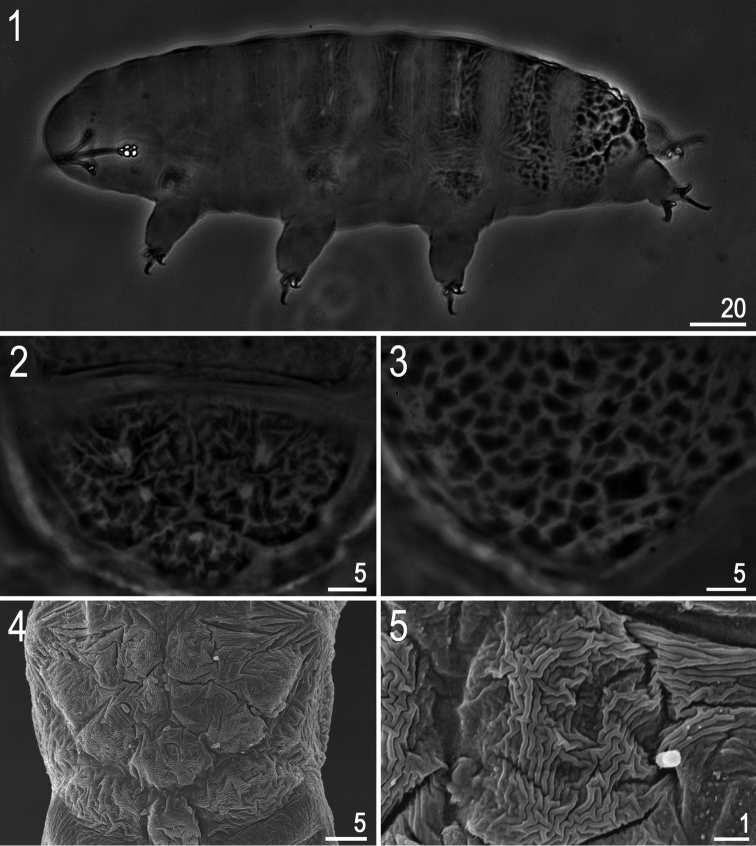
*Hypsibius* cf. *scabropygus* Cuénot, 1929: **1** habitus (dorso-lateral view) **2–4** caudo-dorsal cuticle with distinct sculpturing – tubercles and tubercles merged into platelets **5** a single caudo-dorsal platelet. (**1–3:** PCM, **4–5:** SEM).

**(measurements in Table 1). Adults.** Body transparent/white (after preparation), eyes present in 6 of 15 (40%) specimens mounted in Hoyer’s medium (Fig. 1). Dorsal cuticle sculptured: from head to legs II without tubercles but clearly thickened, from legs II to the caudal end of the body (including legs IV) with irregular tubercles and platelets. Tubercles increasing in size from the anterior to the posterior part of the body, reaching maximum dimensions between legs III and IV, where tubercles sometimes merge and form irregular platelets (Figs 2–5). Irregular tubercles 1.0–6.0 μm in diameter. Ventral cuticle smooth (i.e. without sculpturing). Gibbosities and cuticular pores absent.

**Table 1.** Measurements and *pt* values of selected morphological structures of *Hypsibius* cf. *scabropygus* Cuénot, 1929 mounted in Hoyer’s medium (N – number of specimens/structures measured, RANGE refers to the smallest and the largest structure among all measured specimens; SD – standard deviation).

CHARACTER	N	RANGE						MEAN		SD	
		µm			*pt*			µm	*pt*	µm	*pt*
Body length	14	183	–	293	*808*	–	*1132*	235	*949*	33	*97*
Buccopharyngeal tube											
Buccal tube length	15	22.0	–	28.2		–		24.6	–	2.0	–
Stylet support insertion point	15	12.0	–	15.9	*51.3*	–	*57.1*	13.5	*54.9*	1.1	*1.6*
Buccal tube external width	15	1.5	–	2.0	*6.5*	–	*8.0*	1.8	*7.2*	0.2	*0.5*
Buccal tube internal width	15	0.6	–	0.9	*2.1*	–	*3.4*	0.7	*2.7*	0.1	*0.4*
Placoid lengths											
Macroplacoid 1	15	1.8	–	3.2	*7.7*	–	*12.3*	2.4	*9.6*	0.4	*1.3*
Macroplacoid 2	15	1.7	–	2.7	*7.2*	–	*11.3*	2.2	*8.9*	0.3	*1.1*
Macroplacoid row	15	4.7	–	6.3	*19.1*	–	*26.5*	5.3	*21.7*	0.5	*1.8*
Claw 1 lengths											
External base	14	2.2	–	4.5	*9.1*	–	*17.2*	3.5	*14.1*	0.7	*2.1*
External primary branch	14	4.0	–	8.8	*18.2*	–	*36.1*	6.9	*28.1*	1.5	*5.0*
External secondary branch	14	2.3	–	5.9	*10.5*	–	*24.4*	4.4	*17.9*	1.0	*3.9*
Internal base	12	2.1	–	4.1	*9.3*	–	*15.5*	3.3	*13.4*	0.6	*1.8*
Internal primary branch	12	3.8	–	5.9	*16.0*	–	*23.5*	4.8	*19.3*	0.6	*2.0*
Internal secondary branch	12	2.4	–	4.3	*9.9*	–	*16.5*	3.3	*13.3*	0.6	*2.0*
Claw 2 lengths											
External base	11	3.0	–	5.2	*12.4*	–	*19.9*	4.1	*16.7*	0.7	*2.2*
External primary branch	13	6.7	–	10.4	*29.7*	–	*43.7*	8.5	*34.7*	1.3	*4.4*
External secondary branch	13	4.3	–	6.7	*19.0*	–	*27.2*	5.4	*21.9*	0.7	*2.6*
Internal base	10	2.4	–	4.5	*10.9*	–	*18.9*	3.6	*14.6*	0.7	*2.2*
Internal primary branch	12	4.0	–	6.7	*17.7*	–	*27.2*	5.4	*22.0*	0.9	*2.9*
Internal secondary branch	12	2.6	–	5.4	*11.8*	–	*22.0*	4.1	*16.7*	0.9	*3.0*
Claw 3 lengths											
External base	9	2.7	–	6.2	*11.9*	–	*23.8*	4.3	*17.3*	1.0	*3.5*
External primary branch	9	7.2	–	10.4	*29.3*	–	*43.7*	8.8	*35.7*	1.1	*4.4*
External secondary branch	9	3.6	–	6.5	*12.8*	–	*27.3*	5.2	*21.0*	1.0	*4.3*
Internal base	11	2.3	–	4.1	*10.5*	–	*17.2*	3.4	*13.9*	0.6	*1.9*
Internal primary branch	13	3.8	–	6.5	*17.3*	–	*27.3*	5.4	*21.8*	0.9	*3.1*
Internal secondary branch	12	2.7	–	6.1	*12.2*	–	*24.8*	3.9	*16.0*	0.9	*3.5*
Claw 4 lengths											
Anterior base	13	3.3	–	5.6	*12.8*	–	*20.1*	4.1	*16.8*	0.6	*2.2*
Anterior primary branch	13	4.4	–	7.5	*19.5*	–	*31.1*	5.9	*24.2*	1.1	*3.9*
Anterior secondary branch	11	3.1	–	13.2	*13.0*	–	*47.3*	4.8	*18.9*	2.9	*9.7*
Posterior base	12	2.7	–	5.4	*12.3*	–	*21.5*	4.5	*18.2*	0.9	*3.1*
Posterior primary branch	12	4.9	–	14.9	*22.0*	–	*60.6*	10.3	*41.9*	2.9	*11.2*
Posterior secondary branch	12	4.0	–	6.5	*15.4*	–	*25.6*	5.2	*21.2*	0.9	*3.4*

**Figures 6–7.**
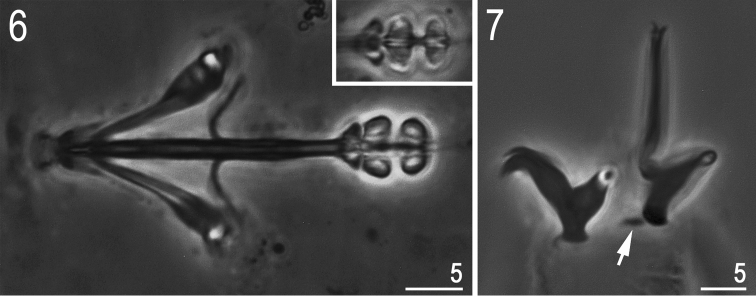
*Hypsibius* cf. *scabropygus* Cuénot, 1929: **6** bucco-pharyngeal apparatus (dorso-ventral projection, ventral placoids in the insert) **7** claws IV (arrow indicates a small cuticular bar near the posterior claw). (Both PCM).

Bucco-pharyngeal apparatus of the *Hypsibius* type, without the ventral lamina, and with forked apophyses for stylet muscles (Fig. 6). Peribuccal lamellae absent. Teeth in the oral cavity armature absent or not visible under PCM. Pharyngeal bulb with apophyses and with two granular macroplacoids (both, without constrictions). Macroplacoid length sequence 2<1. Microplacoid and septulum absent.

Claws of the *Hypsibius* type, internal claws much smaller and of a different shape than the external claws (Fig. 7). All main branches with large accessory points. Smooth, indistinct areoles under claws usually visible only on posterior claws IV. Cuticular bars under claws I-III absent but a small bar is present near the posterior claw IV (Fig. 7, arrow).

**Eggs.** Unknown.

############ Remarks.

*Hypsibius scabropygus* has been recorded from many localities, mostly in the Holarctic (McInnes 1994). In general, our specimens correspond to the original description by Cuénot (1929, 1932) and later descriptions by Marcus (1930) (=*Hypsibius callimerus* spec. nov.), and by Ramazzotti and Maucci (1983). However, importantly, none of the above mentioned descriptions reported a bar between anterior and posterior claws IV, which is present in all our specimens. Given the bar is small, it is possible that it was overlooked by Cuénot and later authors. If, however, *Hypsibius scabropygus* does not have the bar, then our specimens should probably be classified as a new species. Thus, until *Hypsibius scabropygus* is redescribed, our Latvian and Estonian records should be regarded as *Hypsibius* cf. *scabropygus*. As there is a possibility of our specimens belonging to a new species, we provide standard morphometrics (Table 1) and photographs (Figs 1–7).

######### Subfamily: Itaquasconinae Rudescu, 1964

########## Genus: *Astatumen* Pilato, 1997

########### Astatumen bartosi

(Węglarska, 1959)

############ Localities and specimen numbers.

XIV: 1 specimen.

############ Remarks.

Our specimen corresponds perfectly with characters of *Astatumen bartosi* proposed by Dastych (1988) with the main difference between *Astatumen bartosi* and *Astatumen trinacriae* being the absence/presence of cuticular bars on legs II–III. Due to the notorious difficulties in differentiating the two species, the actual distribution of *Astatumen bartosi* cannot currently be described with confidence. McInnes (1994) cited this species from several localities in Europe and from single African, Asian and South American sites.

####### Superfamily: Isohypsibioidea Marley, McInnes & Sands, 2011

######## Family: Isohypsibiidae Marley, McInnes & Sands, 2011

######### Genus: *Isohypsibius* Thulin, 1928

########## Isohypsibius ronsisvallei

Binda & Pilato, 1969

########### Localities and specimen numbers.

III: 1 specimen.

########### Remarks.

The species has previously been reported from several, mostly European, localities in the Holarctic (McInnes 1994).

########## Isohypsibius sattleri

(Richters, 1902)

########### Localities and specimen numbers.

IX: 1 specimen, XI: 8 specimens (including 5 simplexes), XIV: 3 specimens, XV: 5 specimens, XVI: 1 specimen.

########### Remarks.

The species has previously been reported from many localities throughout the World, thus it is considered cosmopolitan (McInnes 1994, Kaczmarek et al. 2014a).

####### Superfamily: Macrobiotoidea Thulin, 1928 in Marley et al. 2011

######## Family: Macrobiotidae Thulin, 1928

######### Genus: *Macrobiotus* C.A.S. Schultze, 1834

########## Macrobiotus harmsworthi harmsworthi

Murray, 1907

########### Localities and specimen numbers.

XI: 1 egg, XIV: 8 specimens, 1 egg.

########### Remarks.

The species belongs to the *harmsworthi* group which is widely distributed across a broad range of ecosystems throughout the world (McInnes 1994, Kaczmarek et al. 2014a). In the last decade many new species within this group were described from a variety of localities (Michalczyk and Kaczmarek 2003b, Pilato et al. 2004, Tumanov 2005a, Pilato and Lisi 2006a, b, Pilato et al. 2006a, Kaczmarek et al. 2007, Kaczmarek and Michalczyk 2009b, Pilato and Lisi 2009a, Rossi et al. 2009, see also Kaczmarek et al. 2011 for the diagnostic key to the group). Due to many uncertain reports of *Macrobiotus harmsworthi harmsworthi*, especially in older literature, the distribution of the species is currently unknown. Specimens found in the present study correspond well to the characters presented in Pilato et al. (2000) and were successfully identified with the key by Kaczmarek et al. (2011).

########## Macrobiotus hufelandi hufelandi

C.A.S. Schultze, 1833

########### Localities and specimen numbers.

XIV: 4 specimens, 1 egg.

########### Remarks.

The species belongs to the *hufelandi* group which is widely distributed across a broad range of ecosystems throughout the world (McInnes 1994, Kaczmarek et al. 2014a). In the last decade new species belonging to this group have been described from various localities (Pilato et al. 2003, Kaczmarek and Michalczyk 2004, Dastych 2002, 2005, Fontoura et al. 2008, Bartels et al. 2009, Kaczmarek and Michalczyk 2009b, Pilato and Lisi 2009b, Bertolani et al. 2011, Biserov et al. 2011, Pilato et al. 2012, Guidetti et al. 2013, see also Bertolani and Rebecchi 1993 for the diagnostic key to the group). Due to many uncertain reports of *Macrobiotus hufelandi hufelandi*, especially in older literature, the distribution of the species is currently unknown. Specimens found in the present study correspond well with the redescription by Bertolani and Rebecchi (1993).

######### Genus: *Minibiotus* R.O. Schuster, 1980

########## Minibiotus formosus sp. n.

http://zoobank.org/BDBE49B7-84CF-4FE2-BE55-A399A537DE77

http://species-id.net/wiki/Minibiotus_formosus

http://www.tardigrada.net/register/0012.htm

Tables 2 –3 , Figs 8 –15

########### Type material.

Holotype and 23 paratypes, 24 specimens (including 2 simplexes) and 3 unembryonated eggs).

########### Type locality.

57°10'33"N, 24°50'32"E, *ca.* 45 m asl: Latvia, Vidzeme region, Sigulda municipality, Gutmana Cave in the Gauja National Park; moss from rock (1 sample, slide codes: ŁO 2427/*, where the asterisk can be substituted by any of the following numbers: 1, 2, 3, 4, 5, 6, 7, 8, 9, 10, 11, 12).

########### Description

**Figures 8–11.**
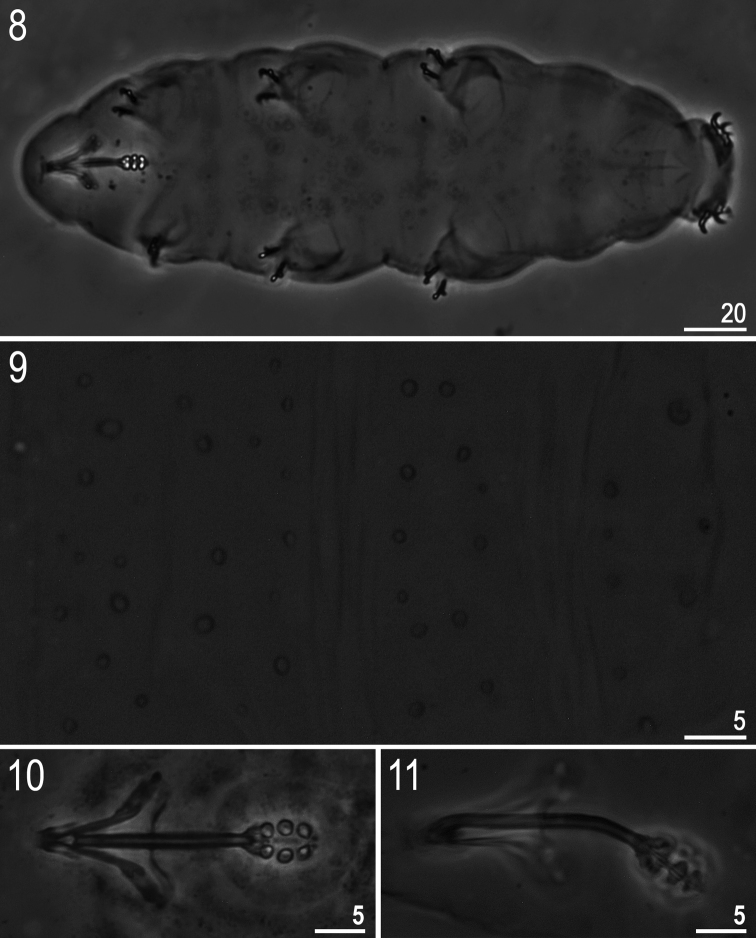
*Minibiotus formosus* sp. n.: **8** habitus (holotype, ventral view) **9** dorsal cuticle with pores (holotype) **10–11** bucco-pharyngeal apparatus (**10** dorso-ventral projection, paratype **11** lateral view, paratype). All PCM.

**Figures 12–15.**
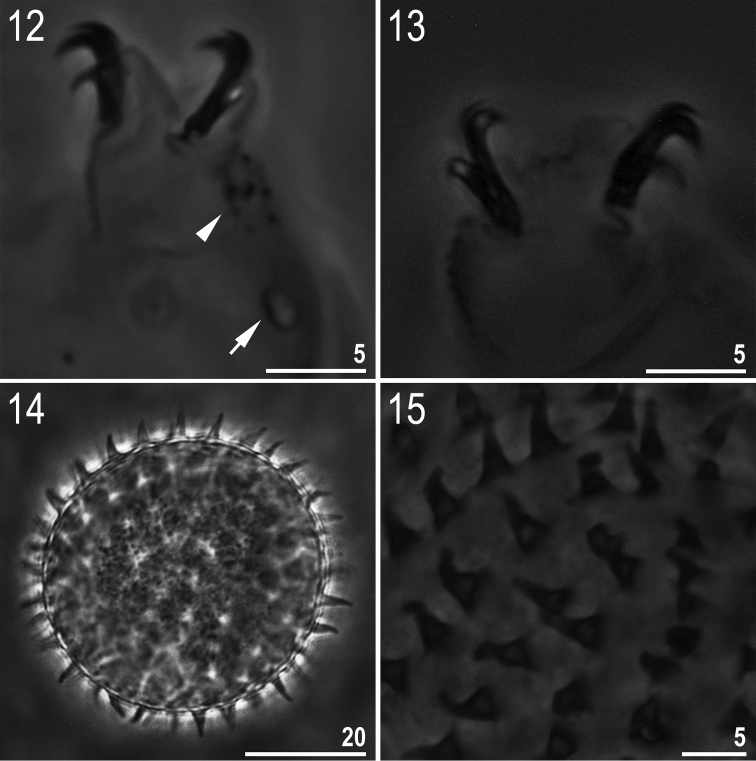
*Minibiotus formosus* sp. n.: **12** leg II with claws, granulation (arrowhead) and a single large pore (arrow) (holotype) **13** claws IV (paratype) **14** egg (mid-section) **15** egg surface with processes. All PCM.

**(measurements in Table 2).** Body white/colourless (Fig. 8). Eyes present in 18 of 24 (75%) specimens mounted in Hoyer’s medium. Entire cuticle covered with small (0.4–1.1 μm) and large (1.9–2.5 μm) round or oval pores (Fig. 9). Pores arranged in 9–10 poorly defined transverse bands. Pores on the dorsal cuticle arranged more densely than on the ventral cuticle. A single large pore (diameter: 2.1–2.9 μm) present on external side of legs I–III (Fig. 12, arrow). A ring of pores around the mouth opening absent. Cuticle without granulation, except for legs which are all covered with fine and regular granulation (better developed on legs IV) visible only in larger specimens (Fig. 12, arrowhead).

**Table 2.** Measurements and *pt* values of selected morphological structures of *Minibiotus formosus* sp. n. mounted in Hoyer’s medium (N – number of specimens/structures measured, RANGE refers to the smallest and the largest structure among all measured specimens; SD – standard deviation, ? – trait oriented unsuitably for measurement).

CHARACTER	N	RANGE						MEAN		SD		Holotype	
		µm			*pt*			µm	*pt*	µm	*pt*	µm	*pt*
Body length	7	113	–	236	636	–	1034	184	901	39	139	194	848
Buccal tube													
Length	9	17.7	–	22.9		–		20.3	–	1.7	–	22.9	–
Stylet support insertion point	9	9.5	–	12.2	49.5	–	56.2	10.9	53.8	0.9	2.0	12.2	53.3
External width	9	1.3	–	2.0	7.0	–	9.9	1.6	7.8	0.2	0.8	1.7	7.4
Internal width	9	0.5	–	0.7	2.4	–	3.4	0.6	2.8	0.1	0.3	0.7	3.1
Placoid lengths													
Macroplacoid 1	9	1.3	–	1.9	6.9	–	8.3	1.5	7.6	0.2	0.4	1.8	7.9
Macroplacoid 2	9	1.2	–	1.7	6.8	–	8.3	1.5	7.5	0.2	0.4	1.7	7.4
Macroplacoid 3	9	1.4	–	2.2	7.4	–	9.6	1.7	8.2	0.3	0.6	1.9	8.3
Microplacoid	8	0.5	–	0.9	2.4	–	4.7	0.7	3.3	0.1	0.8	0.8	3.5
Macroplacoid row	9	4.5	–	6.8	24.3	–	33.7	5.5	26.9	0.7	3.0	5.9	25.8
Placoid row	8	5.2	–	7.9	27.1	–	39.1	6.4	31.0	0.9	3.9	6.7	29.3
Claw 1 lengths													
External primary branch	4	4.7	–	6.5	22.8	–	31.6	5.8	27.7	0.8	3.7	6.2	27.1
External secondary branch	3	3.6	–	4.3	17.5	–	20.9	3.9	19.4	0.4	1.7	?	?
Internal primary branch	6	4.7	–	6.6	24.3	–	30.4	5.7	27.4	0.7	2.4	6.3	27.5
Internal secondary branch	5	3.1	–	4.6	15.0	–	20.4	4.0	18.7	0.5	2.3	4.6	20.1
Claw 2 lengths													
External primary branch	5	5.2	–	6.9	25.2	–	34.0	5.9	28.6	0.6	3.5	5.9	25.8
External secondary branch	4	3.3	–	4.7	16.0	–	23.2	3.9	19.4	0.6	2.9	?	?
Internal primary branch	5	5.2	–	6.4	27.1	–	31.9	6.0	29.7	0.5	2.3	6.2	27.1
Internal secondary branch	3	3.5	–	4.1	18.5	–	20.9	3.9	19.9	0.3	1.3	?	?
Claw 3 lengths													
External primary branch	5	5.1	–	6.9	27.0	–	33.2	6.3	30.6	0.7	2.4	6.9	30.1
External secondary branch	6	3.6	–	4.9	19.0	–	24.1	4.6	22.1	0.5	1.8	4.9	21.4
Internal primary branch	7	5.1	–	6.6	25.7	–	32.5	5.9	29.2	0.6	2.5	6.4	27.9
Internal secondary branch	4	4.1	–	4.6	20.9	–	22.8	4.3	21.6	0.2	0.8	?	?
Claw 4 lengths													
Anterior primary branch	5	6.0	–	8.0	31.6	–	39.6	7.0	34.2	0.8	3.5	?	?
Anterior secondary branch	4	4.0	–	6.0	21.2	–	29.7	5.0	24.1	0.9	3.9	?	?
Posterior primary branch	6	6.0	–	7.8	30.7	–	38.4	7.0	33.9	0.7	3.1	?	?
Posterior secondary branch	5	3.9	–	5.5	20.6	–	27.1	4.8	23.8	0.6	2.6	?	?

Mouth antero-ventral. Ten peribuccal papulae present. Bucco-pharyngeal apparatus of the *Minibiotus* type (Figs 10–11). Oral cavity armature absent or not visible under PCM. Buccal tube with a poorly visible ventral lamina and with an anterior and a posterior bend (both visible in lateral view only, Fig. 11). Buccal tube walls thickened just below the stylet supports insertion point. Pharyngeal apophyses triangular, very near to the first macroplacoid. Three granular macroplacoids and a minute microplacoid present in the pharyngeal bulb. All macroplacoids of similar but not identical sizes, the macroplacoid length sequence: 2<1<3. Septulum absent.

Claws of the *Macrobiotus* type (Figs 12–13). Primary branches of claws with thin, but obvious accessory points detaching at the apogee of the primary branch curve. Smooth lunules present on all legs, distinctly larger under external and posterior claws. Bars and other cuticular thickenings on legs absent.

**Eggs (measurements in Table 3).** White/transparent, laid freely (Fig. 14). Spherical, without areolation. Processes in the shape of short, smooth, slightly flexible cones (Fig. 15). Processes are distributed on the surface of the egg close one to another but never in contact. Surface between processes smooth under PCM (Fig. 15).

**Table 3.** Measurements of selected morphological structures of *Minibiotus formosus* **sp. n.** eggs mounted in Hoyer’s medium.

CHARACTER	egg 1	egg 2	egg 3
Diameter of egg without processes	45.7	44.1	?
Diameter of egg with processes	55.6	55.1	?
Process height	4.5–5.2	4.8–5.2	4.6–5.3
Process base width	2.8–3.4	2.8–3.1	2.4–2.6
Process base/height ratio	57%–69%	54%–65%	47%–57%
Distance between processes	2.0–2.5	1.9–3.9	1.8–2.0
Number of processes on the egg circumference	30	29	30

########### Remarks.

Since ventral lamina is very poorly visible, the measurements of this structure are not included in Table 2. Three unembryonated eggs have been found alongside the described specimens. Given that *Minibiotus formosus* sp. n. was the only *Minibiotus* species in the sample and because no *Ramazzottius* Binda & Pilato, 1986 was found in the samples, we assumed that these eggs belong to the new species.

########### Etymology.

Given that we found the composition of small and large pores in the new species beautiful, we decided to name the animal after this impression (in Latin ‘*formosus*’ means ‘beautiful’).

########### Type depositories.

Holotype 23 paratypes and 3 eggs are deposited in the Department of Animal Taxonomy and Ecology at the Adam Mickiewicz University (Poznań, Poland).

### Differential diagnosis

The new species is most similar to *Macrobiotus gumersindoi* Guil & Guidetti, 2005, but it differs from it by: the presence of two types of cuticular pores (small and large) in the new species *vs* pores of a uniform size in *Macrobiotus gumersindoi*, the absence of a triangular or a pentagonal arrangement of pores placed above a single large pore on legs, the presence of granulation on legs, a different macroplacoid length sequence (2<1<3 in the new species *vs* 1=2=3 in *Macrobiotus gumersindoi*), and by slightly larger macroplacoids (I: 1.3–1.9 μm; II: 1.2–1.7 μm III: 1.4–2.2 μm in the new species *vs* 1.0 μm in *Macrobiotus gumersindoi*).

Other species to which *Minibiotus formosus* sp. n. is similar by some characteristics of adult and/or egg morphology (e.g. pores in transverse bands, eggs with conical processes), include species listed below. The new species differs specifically from:

- ***Macrobiotus bisoctus*** (Horning et al. 1978) by: the absence of trilobed and star-shaped pores (although their presence was not mentioned in the original description, they are clearly visible in Fig. 114 in Horning et al. (1978), and by stylet supports inserted in a more anterior position (*pt=49.5–56.2* in the new species *vs pt≈60.3* in *Macrobiotus bisoctus* (according to Claxton 1998)).
- ***Macrobiotus eichhorni*** Michalczyk & Kaczmarek, 2004 by: a different arrangement of pores on the dorsal cuticle (9–10 transverse bands in the new species *vs* 8 bands in *Macrobiotus eichhorni*), the absence of star-shaped pores, the absence of four pores around the mouth opening, the presence of a single large pore on lateral sides of legs I-III, slightly shorter buccal tube (17.7–22.9 μm in the new species *vs* 24.7–34.2 μm in *Macrobiotus eichhorni*), stylet supports inserted in more anterior position (9.5–12.2 μm [*pt=49.5–56.2*] in the new species *vs* 16.2–23.8 μm [*pt=65.4–70.6*] in *Macrobiotus eichhorni*), a different macroplacoid sequence (2<1<3 in the new species *vs* 2<3<1 μm in *Macrobiotus eichhorni*), slightly shorter placoids, and by slightly smaller external claws I–IV (compare Table 2 below and Table 1 in Michalczyk and Kaczmarek 2004 for exact differences in dimensions of placoids and claws).
- ***Macrobiotus furcatus*** (Ehrenberg, 1859) (according to Binda and Pilato 1992) by: the absence of tri- and quadrilobed cuticular pores, the presence of two types of cuticular pores (small and large in the new species *vs* uniformly small pores present in *Macrobiotus furcatus*), the presence of a single large pore on each of legs I–III, the presence of granulation on legs, the absence of the oral cavity armature, stylet supports inserted in a more anterior position (*pt=49.5*–*56.2* in the new species *vs pt≈68.4* in *Macrobiotus furcatus*), a different macroplacoid length sequence (2<1<3 in the new species *vs* 2<3<1 in *Macrobiotus furcatus*), and by egg processes without an obvious flexible portion (and never bifurcated).
- ***Macrobiotus harrylewisi*** Meyer & Hinton, 2009 by: the absence of tri- and quadrilobed cuticular pores, the presence of two types of pores (small and large) over the entire cuticle in the new species *vs* small pores present only in the anterior part of the body and large pores present only in the posterior part of the body in *Macrobiotus harrylewisi*, the presence of a single large pore on each of legs I–III, stylet supports inserted in a more anterior position (*pt=49.5*–*56.2* in the new species *vs pt=61.4*–*67.6* in *Macrobiotus harrylewisi*), a different macroplacoid length sequence (2<1<3 in the new species *vs* 2≤3<1 μm in *Macrobiotus harrylewisi*), a different shape of egg processes (short, single-tipped cones in the new species *vs* elongated, tapering cones with bulbous bases in *Macrobiotus harrylewisi*), a smaller diameter of eggs without and with processes (44.1–45.7 μm and 55.1–55.6 μm in the new species *vs* 66.1–80.0 μm and 78.2–101.9 μm in *Macrobiotus harrylewisi*), a slightly lower number of processes on egg circumference (29–30 in the new species *vs* 32–41 in *Macrobiotus harrylewisi*), and by smaller egg processes (4.5–5.3 μm in the new species *vs* 7.6–12.8 μm in *Macrobiotus harrylewisi*).
- ***Macrobiotus jonesorum*** Meyer et al., 2011 by: the absence of trilobed and polygonal pores, the presence of two types of cuticular pores (small and large) in the new species *vs* small pores present only in the anterior part of the body, intermediate in size in the middle of the body and large pores in the posterior part of the body in *Macrobiotus jonesorum*), the presence of a single large pore on each of legs I-III, the presence of granulation on all legs, a slightly shorter buccal tube (17.7–22.9 μm in the new species *vs* 24.4–29.6 μm in *Macrobiotus jonesorum*), stylet supports inserted in a more anterior position (*pt=49.5*–*56.2* in the new species *vs pt=63.0*–*65.6* in *Macrobiotus jonesorum*), a slightly smaller external diameter of the buccal tube (1.3–2.0 μm [*pt=7.0-9.9*] in the new species *vs* 2.1–2.6 μm [*pt=7.4*–*10.7*] in *Macrobiotus jonesorum*), a different macroplacoid length sequence (2<1<3 in the new species *vs* 1<2<3 in *Macrobiotus jonesorum*), slightly shorter macroplacoids II and III (II: 1.2–1.7 μm [*pt=6.8*–*8.6*]; III: 1.4–2.2 μm [*pt=7.4*–*9.6*] in the new species *vs* II: 1.9–2.3 μm [*pt=7.1*–*8.8*]; III: 2.4–2.6 μm [*pt=8.4*–*9.9*] in *Macrobiotus jonesorum*), a slightly shorter macroplacoid row (4.5–6.8 μm [*24.3*–*33.7*] in the new species *vs* 7.0–8.4 μm [*pt=27.0*–*34.4*] in *Macrobiotus jonesorum*), the presence of a microplacoid, and by slightly shorter primary and secondary branches of external claws I–IV (compare Table 2 below and Table 2 in Meyer et al. 2011).
- ***Macrobiotus keppelensis*** Claxton, 1998 by: the lack of red pigment granules, the presence of two types of cuticular pores (small and large) in the new species *vs* pores uniform in size (*ca.* 1.0 μm) in *Macrobiotus keppelensis*), the presence of a single large pore on each of legs I-III, a slightly shorter buccal tube (17.7–22.9 μm in the new species *vs* 24.9–28.4 μm in *Macrobiotus keppelensis*), stylet supports inserted in a more anterior position (*pt=49.5*–*56.2* in the new species *vs pt≈60.6* in *Macrobiotus keppelensis*), a different macroplacoid length sequence (2<1<3 in the new species *vs* 2=3<1 in *Macrobiotus keppelensis*), a slightly shorter macroplacoid row (4.5–6.8 μm in the new species *vs* 7.0–7.6 μm in *Macrobiotus keppelensis*), the lack of a membrane around egg processes, a smaller diameter of eggs with processes (55.1–55.6 μm in the new species *vs* 65.0–85.0 μm in *Macrobiotus keppelensis*), a larger number of processes on egg circumference (29–30 in the new species *vs ca.* 11 in *Macrobiotus keppelensis*), smaller egg processes (4.5–5.3 μm in the new species *vs* 11.0–16.0 μm in *Macrobiotus keppelensis*), narrower egg processes bases (2.4–3.4 μm in the new species *vs* 9.0–12.0 μm in *Macrobiotus keppelensis*), and by slightly smaller distances between egg processes (1.8–3.9 μm in the new species *vs* 4.0–6.0 μm in *Macrobiotus keppelensis*).
- ***Macrobiotus orthofasciatus*** Fontoura et al., 2009 by: cuticular pores arranged in 9–10 transverse bands (11 transverse bands present in *Macrobiotus orthofasciatus*), the absence of tri- and quadrilobed cuticular pores, the presence of two types of pores (small and large) in the new species *vs* all pores of similar size in *Macrobiotus orthofasciatus*, the presence of a single large pore on each of legs I–III, the presence of granulation on all legs, stylet supports inserted in a more anterior position (*pt=49.5*–*56.2* in the new species *vs pt=66.5*–*67.8* in *Macrobiotus orthofasciatus*), a different shape of egg processes (short, single tip cones without a membrane in the new species *vs* screw-like processes with a membrane and six areoles in *Macrobiotus orthofasciatus*), a slightly larger number of processes on egg circumference (29–30 in the new species *vs ca.* 24 in *Macrobiotus orthofasciatus*), and by smaller distances between egg processes (1.8–3.9 μm in the new species *vs* 6.4–6.9 μm in *Macrobiotus orthofasciatus*).
- ***Macrobiotus poricinctus*** Claxton, 1998 by: cuticular pores arranged in 9–10 transverse bands (8 transverse bands in *Macrobiotus poricinctus*), the presence of two types of pores (small and large) in the new species *vs* uniform pore size in *Macrobiotus poricinctus*), the presence of a single large pore on each of legs I–III, stylet supports inserted in a more anterior position (*pt=49.5*–*56.2* in the new species *vs pt≈59.5* in *Macrobiotus poricinctus*), a different macroplacoid length sequence (2<1<3 in the new species *vs* 2=3<1 in *Macrobiotus poricinctus*), a different shape of egg processes (short, single-tipped cones without a membrane in the new species *vs.* screw-like processes within a membrane in *Macrobiotus poricinctus*), the absence of granulation on egg shell, a larger number of processes on egg circumference (29–30 in the new species *vs* 18–20 in *Macrobiotus poricinctus*), slightly smaller egg processes (4.5–5.3 μm in the new species *vs* 6.5–7.0 μm in *Macrobiotus poricinctus*), and by smaller distances between egg processes (1.8–3.9 μm in the new species *vs* 6.0–8.0 μm in *Macrobiotus poricinctus*).
- ***Macrobiotus pustulatus*** (Ramazzotti, 1959) by: the absence of triangular and polygonal pores, the presence of two types of cuticular pores (small and large) in the new species *vs* small pores present only in the anterior part of the body, intermediate in size in the middle of the body and the large pores in the posterior part of the body in *Macrobiotus pustulatus*), the presence of a single large pore on each of legs I–III and, egg processes without a filiform bristle.
- ***Macrobiotus ramazzottii*** Binda & Pilato, 1992 by: pores arranged in bands, the presence of two types of pores (small and large) in the new species *vs* universal pores size in *Macrobiotus ramazzottii*), the presence of a single large pore on each of legs I–III, the absence of the oral cavity armature, stylet supports inserted in a more anterior position (*pt=49.5*–*56.2* in the new species *vs pt=68.2*–*68.3* in *Macrobiotus ramazzottii*), a different macroplacoid length sequence (2<1<3 in the new species *vs* 3<2<1 in *Macrobiotus ramazzottii*), and by a lower number of processes on egg circumference (29–30 in the new species *vs ca.* 34–41 in *Macrobiotus ramazzottii*).
- ***Macrobiotus subintermedius*** (Ramazzotti, 1962) by the presence of cuticular pores, the presence of granulation on all legs, and by fully developed lunules (only small open lunules present in *Macrobiotus subintermedius*).
- ***Macrobiotus vinciguerrae*** Binda & Pilato, 1992 by: pores arranged in bands, the absence of tri- and quadrilobed pores, the presence of two types of pores (small and large) in the new species *vs* uniform pore size in *Macrobiotus vinciguerrae*), the presence of a single large pore on each of legs I–III, the absence of the oral cavity armature, a larger mean body size (184 μm in the new species *vs* 380 μm in *Macrobiotus vinciguerrae*), stylet supports inserted in a more anterior position (*pt=49.5*–*56.2* in the new species *vs pt=66.1*–*68.7* in *Macrobiotus vinciguerrae*), a different macroplacoid length sequence (2<1<3 in the new species *vs* 2<3<1 μm in *Macrobiotus vinciguerrae*), a smaller diameter of eggs without and with processes (44.1–45.7 μm and 55.1–55.6 μm in the new species *vs ca.* 76.4 μm and *ca.* 88.0 μm in *Macrobiotus vinciguerrae*), a slightly larger number of processes on egg circumference (29–30 in the new species *vs ca.* 26 in *Macrobiotus vinciguerrae*), egg processes without flexible filaments, smaller egg processes (4.5–5.3 μm in the new species *vs ca.* 8.2 μm in *Macrobiotus vinciguerrae*), and by narrower bases of egg processes (2.4–3.4 μm in the new species *vs ca.* 5.0 μm in *Macrobiotus vinciguerrae*).
- ***Macrobiotus weglarskae*** Michalczyk et al., 2005 by: the absence of bi-, trilobed and star-shaped pores, the presence of two types of pores (small and large) in the new species *vs* uniform pore size in *Macrobiotus weglarskae*), the absence of 3–5 large triangular or irregularly shaped pores on the caudo-dorsal cuticle above hind legs, the presence of a single large pore on each of legs I-III, a different shape of egg processes (short, single tip cones without a membrane in the new species *vs.* screw-like processes within a membrane in *Macrobiotus weglarskae*), a slightly larger number of processes on egg circumference (29–30 in the new species *vs ca.* 24 in *Macrobiotus weglarskae*), and by slightly wider bases of egg processes (2.4–3.4 μm in the new species *vs* 1.6–2.0 μm in *Macrobiotus weglarskae*).
- ***Macrobiotus xavieri*** Fontoura et al., 2009 by: the absence of trilobed pores, the presence of two types of pores (small and large) in the new species *vs* all pores of similar size in *Macrobiotus xavieri*), the presence of a single large pore on each of legs I–III, the presence of granulation on all legs, a smaller body size (113–236 μm in the new species *vs* 275–410 μm in *Macrobiotus xavieri*), stylet supports inserted in a more anterior position (*pt=49.5*–*56.2* in the new species *vs pt=66.1*–*67.9* in *Macrobiotus xavieri*), a different macroplacoid length sequence (2<1<3 in the new species *vs* 2<3<1 in *Macrobiotus xavieri*). shorter macroplacoids (I: 1.3–1.9 μm [*pt=6.9*–*8.3*]; II: 1.2–1.7 μm [*pt=6.8*–*8.6*] III: 1.4–2.2 μm [*pt=7.4*–*9.6*] in the new species *vs* I: 3.6–4.5 μm [*12.7*–*13.8*]; II: 2.9–3.6 μm [*10.3*–*11.1*] III: 3.0–3.9 μm [*pt=10.9*–*11.9*] in *Macrobiotus xavieri*), a shorter microplacoid (0.5–0.9 μm [*pt=2.4*–*4.7*] in the new species *vs* 1.5–2.0 [*5.0*–*6.2*] in *Macrobiotus xavieri*), a shorter macroplacoid row (4.5–6.8 μm [*pt=24.3*–*33.7*] in the new species *vs* 9.8–12.6 μm [*pt=35.6*–*38.5*] in *Macrobiotus xavieri*), a shorter placoid row (5.2–7.9 μm [*pt=27.1*–*39.1*] in the new species *vs* 10.9–13.9 μm [*39.6*–*43.3*] in *Macrobiotus xavieri*), a different shape of egg processes (short, single-tipped cones in the new species *vs* long cones with bi- or multi-tipped tips in *Macrobiotus xavieri*), egg shell and processes without granulation, a smaller diameter of eggs without and with processes (44.1–45.7 μm and 55.1–55.6 μm in the new species *vs* 56.0–79.0 μm and 80.0–99.2 μm in *Macrobiotus xavieri*), a larger number of processes on egg circumference (29–30 in the new species *vs* 20–23 in *Macrobiotus xavieri*), smaller egg processes (4.5–5.3 μm in the new species *vs* 10.6–19.0 μm in *Macrobiotus xavieri*), and by slightly narrower bases of egg processes (2.4–3.4 μm in the new species *vs* 3.7–6.6 μm in *Macrobiotus xavieri*).

#### Genus: *Paramacrobiotus* Guidetti, Schill, Bertolani, Dandekar & Wolf, 2009

##### Paramacrobiotus richtersi

(Murray, 1911)

###### Localities and specimen numbers.

XI: 2 specimens (including 1 simplex) and 1 egg.

###### Remarks.

*Paramacrobiotus* species (until recently a collection of species within *Macrobiotus*) can be divided into three groups: *areolatus*, *huziori* and *richtersi*, with respect to the combination of two traits: the presence/absence of the microplacoid in the pharynx and the type of egg areolation. *Paramacrobiotus richtersi*, considered cosmopolitan, is recognised as the nominal species for a group of very similar taxa that require careful taxonomic examination of adults and egg morphology for correct identification. In the last decade many new species of this group have been described from various localities (e.g. Pilato et al. 2004, Kaczmarek et al. 2005, Tumanov 2005b, Michalczyk and Kaczmarek 2006a, b, Michalczyk et al. 2006, Pilato et al. 2006a, b, Degma et al. 2008, Bartels et al. 2009, Pilato et al. 2012), with more recent additions including molecular data (Guidetti et al. 2009, Schill et al. 2010).

## Supplementary Material

XML Treatment for Milnesium asiaticum

XML Treatment for Milnesium tardigradum tardigradum

XML Treatment for Diphascon (Diphascon) bullatum

XML Treatment for Diphascon (Diphascon) pingue pingue

XML Treatment for Diphascon (Diphascon) recamieri

XML Treatment for Diphascon (Diphascon) rugosum

XML Treatment for Diphascon (Adropion) prorsirostre

XML Treatment for Hypsibius convergens

XML Treatment for Hypsibius dujardini

XML Treatment for Hypsibius cf. scabropygus

XML Treatment for Astatumen bartosi

XML Treatment for Isohypsibius ronsisvallei

XML Treatment for Isohypsibius sattleri

XML Treatment for Macrobiotus harmsworthi harmsworthi

XML Treatment for Macrobiotus hufelandi hufelandi

XML Treatment for Minibiotus formosus

XML Treatment for Paramacrobiotus richtersi

## References

[B1] Argue CW (1974) Tardigrades from New Brunswick, Canada. 3. Canadian Journal of Zoology 52: 919-992. doi: 10.1139/z74-122 10.1139/z71-060 5575665

[B2] Bartels PJ Pilato G Lisi O Nelson DR (2009) *Macrobiotus* (Eutardigrada, Macrobiotidae) from the Great Smoky Mountains National Park, Tennessee/North Carolina, USA (North America): two new species and six new records. Zootaxa 202: 45-57

[B3] Beasley CW Kaczmarek Ł Michalczyk Ł (2008) *Doryphoribius mexicanus*, a new species of Tardigrada (Eutardigrada: Hypsibiidae) from Mexico (North America). Proceedings of the Biological Society of Washington 121(1): 34-40. doi: 10.2988/07-30.1

[B4] Bertolani R Rebecchi L (1993) A revision of the *Macrobiotus hufelandi* group (Tardigrada, Macrobiotidae), with some observations on the taxonomic characters of eutardigrades. Zoologica Scripta 22: 127-152. doi: 10.1111/j.1463-6409.1993.tb00347.x

[B5] Bertolani R Rebecchi L Giovannini I Cesari M (2011) DNA barcoding and integrative taxonomy of *Macrobiotus hufelandi* C.A.S. Schultze 1834, the first tardigrade species to be described, and some related species. Zootaxa 2997: 19-36

[B6] Binda MG Pilato G (1992) *Minibiotus furcatus*, nuova posizione sistematica per *Macrobiotus furcatus* Ehrenberg, 1859, e descrizione di due nuove specie. Animalia 19: 111-120

[B7] Biserov VI Pilato G Lisi O (2011) *Macrobiotus turnovae* sp. n., a new species of tardigrade from Russia. Invertebrate Zoology 8(1): 57-62

[B8] Cuénot L (1929) Description d’un tardigrade nouveau de la faune francaise. Archives d’anatomie Microscopique 25: 121-125

[B9] Cuénot L (1932) Tardigrades. In: Lechevalier P (Ed) Faune de France 24: 1-96

[B10] Claxton SK (1998) A revision of the genus *Minibiotus* (Tardigrada: Macrobiotidae) with descriptions of eleven new species from Australia. Records of the Australian Museum 50: 125-160. doi: 10.3853/j.0067-1975.50.1998.1276

[B11] Dastych H (1980) Niesporczaki (Tardigrada) Tatrzańskiego Parku Narodowego. Monografie Fauny Polski 9: 1-232

[B12] Dastych H (1988) The Tardigrada of Poland. Monografie Fauny Polski 16: 1-255

[B13] Dastych H (1990) *Isohypsibius sattleri* (Richters 1902), a valid species (Tardigrada). Senckenbergiana Biologica 71: 181-189

[B14] Dastych H (2002) A new species of the genus *Macrobiotus* Schultze, 1834 from Iles Kerguélen, in the sub-Antarctic (Tardigrada). Mitteilungen aus dem Hamburgischen Zoologischen Museum und Institut 99: 11-27

[B15] Dastych H (2005) *Macrobiotus ramoli* sp. n., a new tardigrade species from the nival zone of the Ötztal Alps, Austria (Tardigrada). Mitteilungen aus dem Hamburgischen Zoologischen Museum und Institut 102: 21-35

[B16] Degma P Bertolani R Guidetti R (2013) Actual checklist of Tardigrada species. Ver. 23: 15–07–2013 http://www.tardigrada.modena.unimo.it/miscellanea/Actual%20checklist%20of%20Tardigrada.pdf

[B17] Degma P Michalczyk Ł Kaczmarek Ł (2008) *Macrobiotus derkai*, a new species of Tardigrada (Eutardigrada, Macrobiotidae, *huzori* group) from the Colombian Andes (South America). Zootaxa 1731: 1-23

[B18] Ehrenberg CG (1859) Beitrag zur Bestimmung des stationären mikroscopischen Lebens in bis 20,000 Fuss Alpenhöhe. Abhandlungen der Königlichen Akademie der Wissenschaften in Berlin 1858: 429-456

[B19] Fontoura P Pilato G (2007) *Diphascon (Diphascon) faialense* sp. n. a new species of Tardigrada (Eutardigrada, Hypsibiidae) from Azores and a key to the species of the *D. pingue* group. Zootaxa 1589: 47-55

[B20] Fontoura P Pilato G Lisi O (2008) New records of eutardigrades (Tardigrada) from Faial and Pico Islands, the Azores, with the description of two new species. Zootaxa 1778: 37-47

[B21] Fontoura P Pilato G Lisi O Morais P (2009a) Tardigrades from Portugal: four new records and description of two new species. Zootaxa 2030: 21-38

[B22] Fontoura P Pilato G Morais P Lisi O (2009b) *Minibiotus xavieri*, a new species of tardigrade from Parque Biológico de Gaia, Portugal (Eutardigrada: Macrobiotidae). Zootaxa 2267: 55-64

[B23] Guidetti R Bertolani R Degma P (2007) New taxonomic position of several *Macrobiotus* species (Eutardigrada: Macrobiotidae). Zootaxa 1471: 61-68

[B24] Guidetti R Schill RO Bertolani R Dandekar T Wolf M (2009) New molecular data for tardigrade phylogeny, with the erection of *Paramacrobiotus* gen. nov. Journal of Zoological Systematics and Evolutionary Research 47: 315-321. doi: 10.1111/j.1439-0469.2009.00526.x

[B25] Guidetti R Peluffo JR Rocha AM Cesari M Moly de Peluffo MC (2013) The morphological and molecular analyses of a new South American urban tardigrade offer new insights on the biological meaning of the *Macrobiotus hufelandi* group of species (Tardigrada: Macrobiotidae). Journal of Natural History. doi: 10.1080/00222933.2013.800610

[B26] Guil N Guidetti R (2005) A new species of Tardigrada (Eutardigrada: Macrobiotidae) from Iberian Peninsula and Canary Islands (Spain). Zootaxa 889: 1-11

[B27] Holt BG Lessard J-P Borregaard KM Fritz SA Araújo MB Dimitrov D Fabre P-H Graham CH Graves GR Jønsson KA Nogués-Bravo D Wang Z Whittaker RJ Fjeldså J Rahbek C (2012) An Update of Wallace’s Zoogeographic Regions of the World. Science 339(6115): 74-78. doi: 10.1126/science.1228282 23258408 10.1126/science.1228282

[B28] Horning DS Schuster RO Grigarick AA (1978) Tardigrada of New Zealand. New Zealand Journal of Zoology 5: 185-280. doi: 10.1080/03014223.1978.10428316

[B29] Kaczmarek Ł Michalczyk Ł (2004) New records of Tardigrada from Cyprus with a description of the new species *Macrobiotus marlenae* (*hufelandi* group) (Eutardigrada: Macrobiotidae). Genus 15(1): 141-152

[B30] Kaczmarek Ł Michalczyk Ł (2009a) Redescription of *Hypsibius microps* Thulin, 1928 and *H. pallidus* Thulin, 1911 (Eutardigrada: Hypsibiidae) based on the type material from the Thulin collection. Zootaxa 2275: 60-68

[B31] Kaczmarek Ł Michalczyk Ł (2009b) Two new species of Macrobiotidae, *Macrobiotus szeptyckii* (*harmsworthi* group) and *Macrobiotus kazmierskii* (*hufelandi* group) from Argentina. Acta Zoologica Cracoviensia 52B: 87–99. doi: 10.3409/azc.52b_1-2.87-99

[B32] Kaczmarek Ł Michalczyk Ł Degma P (2007) Description of a new tardigrade, *Macrobiotus barbarae* (Eutardigrada: Macrobiotidae), from the Dominican Republic. Annales Zoologici 57(3): 363-369

[B33] Kaczmarek Ł Michalczyk Ł Diduszko D (2005) Some tardigrades from Siberia (Russia, Baikal region) with a description of *Macrobiotus garynahi* sp. n. (Eutardigrada: Macrobiotidae: richtersi group). Zootaxa 1053: 35-45

[B34] Kaczmarek Ł Michalczyk Ł McInnes SJ (2014a) Annotated zoogeography of non-marine Tardigrada. Part I: Central America. Zootaxa 3763(1): 1-62. doi: 10.11646/zootaxa.3763.1.1 24870276 10.11646/zootaxa.3763.1.1

[B35] Kaczmarek Ł Cytan J Zawierucha K Diduszko D Michalczyk Ł (2014b) Tardigrades from Peru (South America), with descriptions of three new species of Parachela. Zootaxa 3790(2): 357-379. doi: 10.11646/zootaxa.3790.2.5 24869872 10.11646/zootaxa.3790.2.5

[B36] Kaczmarek Ł Gołdyn B Prokop ZM Michalczyk Ł (2011) New records of Tardigrada from Bulgaria with the description of *Macrobiotus binieki* sp. n. (Eutardigrada: Macrobiotidae) and a key to the species of the *harmsworthi* group. Zootaxa 2781: 29-39

[B37] Kaczmarek Ł Zawierucha K Smykla J Michalczyk Ł (2012) Tardigrada of the Revdalen (Spitsbergen) with the descriptions of two new species: *Bryodelphax parvuspolaris* (Heterotardigrada) and *Isohypsibius coulsoni* (Eutardigrada). Polar Biology 35(7): 1013-1026. doi: 10.1007/s00300-011-1149-0

[B38] Li X Wang D Wang L (2008) The Tardigrada fauna of Hainan Island (Asia: China) with descriptions of two new species. Raffles Bulletin of Zoology 56(2): 293-305

[B39] Marcus E (1930) Beiträge zur Tardigradensystematik. Zoologische Jahrbuecher Systematik 59: 363-386

[B40] Marley NJ McInnes SJ Sands CJ (2011) Phylum Tardigrada: A re-evaluation of the Parachela. Zootaxa 2819: 51-64

[B41] McInnes SJ (1994) Zoogeographic distribution of terrestrial/freshwater tardigrades from current literature. Journal of Natural History 28: 257-352. doi: 10.1080/00222939400770131

[B42] Meyer HA (2012) A new water bear, *Minibiotus julianae*, from the Caribbean Island of Dominica (Tardigrada: Eutardigrada: Parachela: Macrobiotidae). Proceedings of the Biological Society of Washington 125(1): 54-60. doi: 10.2988/11-15.1

[B43] Meyer HA Domingue MD (2011) *Minibiotus acadianus* (Eutardigrada: Macrobiotidae), a new species of Tardigrada from southern Louisiana, USA. Western North American Naturalist 71(1): 38-43. doi: 10.3398/064.071.0106

[B44] Meyer HA Hinton JG (2009) The Tardigrada of southern Africa, with the description of *Minibiotus harrylewisi*, a new species from KwaZulu-Natal, South Africa (Eutardigrada: Macrobiotidae). African Invertebrates 50(2): 255-268. doi: 10.5733/afin.050.0203

[B45] Meyer HA Lyons AM Nelson DR Hinton JG (2011) Tardigrada of Michigan, Northern USA, with the description of *Minibiotus jonesorum* sp. n. (Eutardigrada: Macrobiotidae). Journal of Zoological Systematics and Evolutionary Research 49(S1): 40–47. doi: 10.1111/j.1439-0469.2010.00596.x

[B46] Michalczyk Ł Kaczmarek Ł (2003a) *Minibiotus constellatus*, a new species of Tardigrada (Eutardigrada, Macrobiotidae) from Peru. Genus 14(2): 295-305

[B47] Michalczyk Ł Kaczmarek Ł (2003b) A description of the new tardigrade *Macrobiotus reinhardti* (Eutardigrada, Macrobiotidae, *harmsworthi* group) with some remarks on the oral cavity armature within the genus *Macrobiotus* Schultze. Zootaxa 331: 1-24

[B48] Michalczyk Ł Kaczmarek Ł (2004) *Minibiotus eichhorni* sp. n., a new species of eutardigrade (Eutardigrada: Macrobiotidae) from Peru. Annales Zoologici 54: 673-676

[B49] Michalczyk Ł Kaczmarek Ł (2006a) *Macrobiotus huziori*, a new species of Tardigrada (Eutardigrada: Macrobiotidae) from Costa Rica (Central America). Zootaxa 1169: 47-59

[B50] Michalczyk Ł Kaczmarek Ł (2006b) A new species *Macrobiotus magdalenae* (Tardigrada: Eutardigrada: Macrobiotidae, *richtersi* group) from Costa Rican rain forest (Central America). New Zealand Journal of Zoology 33: 189-196. doi: 10.1080/03014223.2006.9518444

[B51] Michalczyk Ł Kaczmarek Ł (2013) The Tardigrada Register: a comprehensive online data repository for tardigrade taxonomy. Journal of Limnology 72(S1): 175–181. doi: 10.4081/jlimnol.2013.s1.e22

[B52] Michalczyk Ł Kaczmarek Ł Claxton SK (2005) *Minibiotus weglarskae*, a new species of Tardigrada (Eutardigrada: Macrobiotidae) from Mongolia. Zootaxa 1008: 47-56

[B53] Michalczyk Ł Kaczmarek Ł Weglarskae B (2006) *Macrobiotus sklodowskae* sp. n. (Tardigrada: Eutardigrada: Macrobiotidae, richtersi group) from Cyprus. Zootaxa 1371: 45-46

[B54] Michalczyk Ł Wełnicz W Frohme M Kaczmarek Ł (2012a) Redescriptions of three *Milnesium* Doyère, 1840 taxa (Tardigrada: Eutardigrada: Milnesiidae), including the nominal species for the genus. Zootaxa 3154: 1-20

[B55] Michalczyk Ł Wełnicz W Frohme M Kaczmarek Ł (2012b) Corrigenda of Zootaxa 3154: 1–20 Redescriptions of three *Milnesium* Doyère, 1840 taxa (Tardigrada: Eutardigrada: Milnesiidae), including the nominal species for the genus. Zootaxa 3393: 66-68

[B56] Miller WR McInnes SJ Bergstrøm, DM (2005) Tardigrades of the Australian Antarctic: *Hypsibius heardensis* (Eutardigrada: Hypsibiidae: *dujardini* group) a new species from sub-Antarctic Heard Island. Zootaxa 1022: 57-64

[B57] Murray J (1905) The Tardigrada of the Forth Valley. The Annals of Scottish Natural History 55: 160-164

[B58] Pilato G (1981) Analisi di nuovi caratteri nello studio degli Eutardigradi. Animalia 8: 51-57

[B59] Pilato G (1987) Revision of the genus *Diphascon* Plate, 1889, with remarks on the subfamily Itaquasconinae (Eutardigrada, Hypsibiidae). In: Bertolani (Ed) Biology of Tardigrades Selected Symposia and Monographs U.Z.I., 1: 337–357

[B60] Pilato G (2013) The taxonomic value of the structures for the insertion of the stylet muscles in the Eutardigrada, and description of a new genus. Zootaxa 3721(4): 365-378. doi: 10.11646/zootaxa.3721.4.4 10.11646/zootaxa.3721.4.4 26120681

[B61] Pilato G Binda MG (1997/1998) A comparison of *Diphascon (D.) alpinum* Murray, 1906, *D. (D.) chilenense* Plate, 1889 and *D. (D.) pingue* Marcus, 1936 (Tardigrada), and description of a new species. Zoologischer Anzeiger 236: 181-185

[B62] Pilato G Binda MG (1999) Three new species of *Diphascon* of the *pingue* group (Eutardigrada, Hypsibiidae) from Antarctica. Polar Biology 21: 335-342. doi: 10.1007/s003000050370

[B63] Pilato G Claxton SK (1988) Tardigrades from Australia. 1. *Macrobiotus hieronimi* and *Minibiotus maculartus*, two new species of eutardigrades. Animalia 15: 83-89

[B64] Pilato G Lisi O (2006a) *Macrobiotus rigidus* sp. n., a new species of eutardigrade from New Zealand. Zootaxa 1109: 49-55

[B65] Pilato G Lisi O (2006b) Notes on some tardigrades from southern Mexico with description of three new species. Zootaxa 1236: 53-68

[B66] Pilato G Lisi O (2009a) Tardigrades of the Seychelles Islands, with the description of three new species. Zootaxa 2124: 1-20

[B67] Pilato G Lisi O (2009b) Description of three new species of Tardigrada from Seychelles. Zootaxa 2005: 24-34

[B68] Pilato G Binda MG Lisi O (2003) Remarks on some species of tardigrades from South America with the description of *Minibiotus sidereus* n. sp. Zootaxa 195: 1-8

[B69] Pilato G Binda MG Lisi O (2004) Notes on tardigrades of the Seychelles with the description of three new species. Italian Journal of Zoology 71: 171-178. doi: 10.1080/11250000409356569

[B70] Pilato G Binda MG Lisi O (2006a) Three new species of eutardigrades from the Seychelles. New Zealand Journal of Zoology 33: 39-48. doi: 10.1080/03014223.2006.9518429

[B71] Pilato G Binda MG Lisi O (2006b) Eutardigrada from New Zealand, with descriptions of two new species. New Zealand Journal of Zoology 33: 49-63. doi: 10.1080/03014223.2006.9518430

[B72] Pilato G Binda MG Napolitano A Moncada E (2000) The specific value of *Macrobiotus coronatus* DeBarros 1942, and description of two new species of the *harmsworthi* group (Eutardigrada). Bollettino delle sedute della Accademia Gioenia di Scienze Naturali in Catania 33: 103-120

[B73] Pilato G Kaczmarek Ł Michalczyk Ł Lisi O (2003) *Macrobiotus polonicus*, a new species of Tardigrada from Poland (Eutardigrada, Macrobiotidae, ‘*hufelandi* group’). Zootaxa 258: 1-8

[B74] Pilato G Kiosya Y Lisi O Sabella G (2012) New records of Eutardigrada from Belarus with the description of three new species. Zootaxa 3179: 39-60

[B75] Ramazzotti G (1959) Il gruppo dell’ *Echiniscus viridis* con la nuova species *E. perviridis* e *Macrobiotus pustulatus* altra nuova specie (Tardigrada). Atti della Societa ltaliana di Scienze Naturali e del Museo Civico di Storia Naturale in Milano 98: 303-309

[B76] Ramazzotti G (1962) Tardigradi del Cile, con descrizione di quattro nuove specie e di una varietá. Atti della Società Italiana di Scienze Naturali e del Museo Civico di Storia Naturale di Milano 101: 275-287

[B77] Ramazzotti G Maucci W (1983) Il Phylum Tardigrada. Memorie dell’Istituto Italiano di Idrobiologia 41: 1-1012

[B78] Rossi G Claps M Ardohain D (2009) Tardigrades from northwestern Patagonia (Neuquén Province, Argentina) with the description of three new species. Zootaxa 2095: 21-36

[B79] Šatkauskienė I Vosyliūtė R (2010) Microfauna of Moss (Bryophyta: Bryopsida) from four regions of Lithuania. Acta Zoologica Lituanica 20: 225-231. doi: 10.2478/v10043-010-0024-5

[B80] Schill RO Forster F Dandekar T Wolf N (2010) Using compensatory base change analysis of internal transcribed spacer 2 secondary structures to identify three new species in *Paramacrobiotus* (Tardigrada). Organisms Diversity & Evolution 10(4): 287-296. doi: 10.1007/s13127-010-0025-z

[B81] Tumanov DV (2005a) Two new species of *Macrobiotus* (Eutardigrada, Macrobiotidae) from Tien Shan (Kirghizia), with notes on *Macrobiotus tenuis* group. Zootaxa 1043: 33-46

[B82] Tumanov DV (2005b) Notes on the Tardigrada of Thailand, with description of *Macrobiotus alekseevi* sp. n. (Eutardigrada, Macrobiotidae). Zootaxa 999: 1-16

[B83] Tumanov DV (2006) Five new species of the genus *Milnesium* (Tardigrada, Eutardigrada, Milnesiidae). Zootaxa 1122: 1-23

[B84] Zawierucha K Kaźmierski A (2012) The first records of tardigrades (Tardigrada, Eutardigrada) from Estonia. Zoology and Ecology 22: 111-113. doi: 10.1080/21658005.2012.696885

[B85] Ziemelis A Purina L Ozolinš A (2012) A Short-term study of population Dynamics of tardigrades in the moss *Leucodon sciuroides* (Hedw.) SCHWÄGR. Latvijas Universitātes 70 zinātniskā konference Bioloăijas sekcija, Zooloăijas un dzīvnieku ekoloăijas apakšsekcija (conference abstract).

